# Exploring the role of Tibetan medicinal formula Qishiwei Zhenzhu Pills (Ranasampel) against diabetes mellitus-linked cognitive impairment of db/db mice through serum pharmacochemistry and microarray data analysis

**DOI:** 10.3389/fnagi.2022.1033128

**Published:** 2022-12-22

**Authors:** Zhiyi Yan, Yonghua Zong, Chengfei Zhang, Zekun Han, Lili Wu, Lingling Qin, Tonghua Liu

**Affiliations:** ^1^Dongfang Hospital, Beijing University of Chinese Medicine, Beijing, China; ^2^Key Laboratory of Health-Cultivation, Ministry of Education of the People’s Republic of China, Beijing University of Chinese Medicine, Beijing, China; ^3^Department of Tibetan Medicine, University of Tibetan Medicine, Lhasa, China

**Keywords:** Qishiwei Zhenzhu Pills (Ranasampel), traditional Tibetan medicine, serum pharmacochemistry, microarray data analysis, diabetes cognitive impairment, hippocampus

## Abstract

**Background:**

Diabetes cognitive impairment (DCI) is a common diabetic central nervous system disorder that severely affects the quality of life of patients. Qishiwei Zhenzhu Pills (Ranasampel) is a valuable Tibetan medicine formula with the ability to improve cerebral blood vessels, protect nerves and improve learning and memory, which has also been widely verified in clinical and basic research. Currently, the prevention and treatment of DCI are still in the exploratory research stage, and the use of Ranasampel will provide new ideas and insights for its treatment.

**Objective:**

This study is to explore the absorbed components in serum derived from Ranasampel using serum pharmacochemistry, then identify the potential mechanism of Ranasampel for the treatment of DCI through bioinformatics and microarray data validation.

**Methods:**

The UPLC-Q-Exactive MS/MS-based serum pharmacochemistry method was conducted to identify the main active components in serum containing Ranasampel. Then, these components were used to predict the possible biological targets of Ranasampel and explore the potential targets in treating DCI by overlapping with differentially expressed genes (DEGs) screened from Gene Expression Omnibus datasets. Afterward, the protein–protein interaction network, enrichment analyses, hub gene identification, and co-expression analysis were used to study the potential mechanism of Ranasampel. Particularly, the hub genes and co-expression transcription factors were further validated using hippocampal expression profiles of db/db mice treated with Ranasampel, while the Morris water-maze test and H&E staining were used to assess the spatial learning and memory behaviors and histopathological changes.

**Results:**

Totally, 40 compounds derived from Ranasampel had been identified by serum sample analysis, and 477 genes related to these identified compounds in Ranasampel, 110 overlapping genes were collected by the intersection of Ranasampel target genes and DEGs. Further comprehensive analysis and verification emphasized that the mechanism of Ranasampel treatment of DCI may be related to the improvement of learning and memory function as well as insulin resistance, hyperglycemia-induced neuronal damage, and neuroinflammation.

**Conclusion:**

This study provided useful strategies to explore the potential material basis for compound prescriptions such as Ranasampel. These hub genes and common pathways also provided new ideas for further study of therapeutic targets of DCI and the pharmacological mechanism of Ranasampel.

## 1 Introduction

Diabetes has become an increasingly serious global problem, the global prevalence of which has continued to grow in recent years, reaching 463 million people aged 20−79 in 2019, and expecting to reach 629 million by 2045 (Saeedi et al., 2019). As we all know, the most terrible thing about diabetes is its complications. The complications caused by diabetes are not only difficult to cure but also seriously affect the quality of life of patients and add a heavy burden to families and the social economy (Demir et al., 2021). Neurological impairment is a common chronic complication of diabetes, and the combination of type 2 diabetes and cognitive dysfunction is also very common in clinical practice (Kullmann et al., 2020). Recent epidemiological studies have shown that diabetes significantly increases the risk of Alzheimer’s disease (AD), suggesting a possible correlation between diabetes and the pathogenesis of AD (Baglietto-Vargas et al., 2016). At the same time, many molecular mechanisms and pathological manifestations of diabetes may overlap with factors leading to AD, and common diabetic symptoms such as hyperglycemia, altered insulin signaling, advanced glycosylation, and chronic low-grade inflammation are common underlying mechanisms of vascular and neurological degeneration, as also found in modern studies (Pugazhenthi et al., 2017; Srikanth et al., 2020). In addition to increasing the risk of cerebrovascular disease and stroke by severely affecting cardiovascular health, hyperglycemia also increases the risk of neurodegeneration through a mechanism related to amyloid (Moran et al., 2015; Takenoshita et al., 2018). Furthermore, central neural circuits respond to insulin-related signals that not only enhance systemic insulin sensitivity, regulate peripheral metabolism, and inhibit endogenous gluconeogenesis, but also modulate the cognitive function and appetite to suppress food intake, yet these response mechanisms are impaired in individuals who develop insulin resistance, suggesting that disturbances in insulin signaling represent a potential link between metabolism and cognitive function (Cai et al., 2018; Manaserh et al., 2020).

Traditional Tibetan medicine has a long history of over 2000 years, which is the second-largest traditional medicine system in China and one of the oldest known medical systems in the world (Dakpa, 2014). It combines theories from early Chinese medicine, Indian medicine, and Arabic medicine, then gradually developed into a unique theory and medical system (Li Q. et al., 2018). Qishiwei Zhenzhu Pills (Ranasampel), first recorded in the *Four Medical Tantras* (Luo et al., 2015), has the therapeutic effect of calming, tranquilizing, restoring consciousness and inducing resuscitation, dredging channels and activating collaterals, harmonizing the qi and blood, which is the most prestigious large group of Tibetan medicinal formula used in the treatment of cardiovascular and cerebrovascular diseases in clinical practice (Xu et al., 2020, 2022). At present, there are no clear and well-defined protocols for the management of cognitive dysfunction in diabetes (Chow et al., 2022). Given those diabetic patients with comorbid cognitive impairment tend to be frailer, it is even more important to avoid under or over-treatment, and there is no evidence that intensive glycemic control or the use of specific anti-diabetic drugs can prevent cognitive decline (Draznin et al., 2022). Glucose-lowering drugs are often unable to prevent and treat the risk of cognitive dysfunction, while traditional Chinese medicine, with its multi-targeted, multi-pathway, and precise efficacy, has a relative advantage (Palleria et al., 2016; Chi et al., 2020). Ranasampel has been proven in clinical and experimental studies to improve cerebrovascular, neuroprotective and learning and memory abilities, which could be used in the treatment of cognitive dysfunction and other related disorders (Wu et al., 2016; Zhao et al., 2022).

**FIGURE 1:**
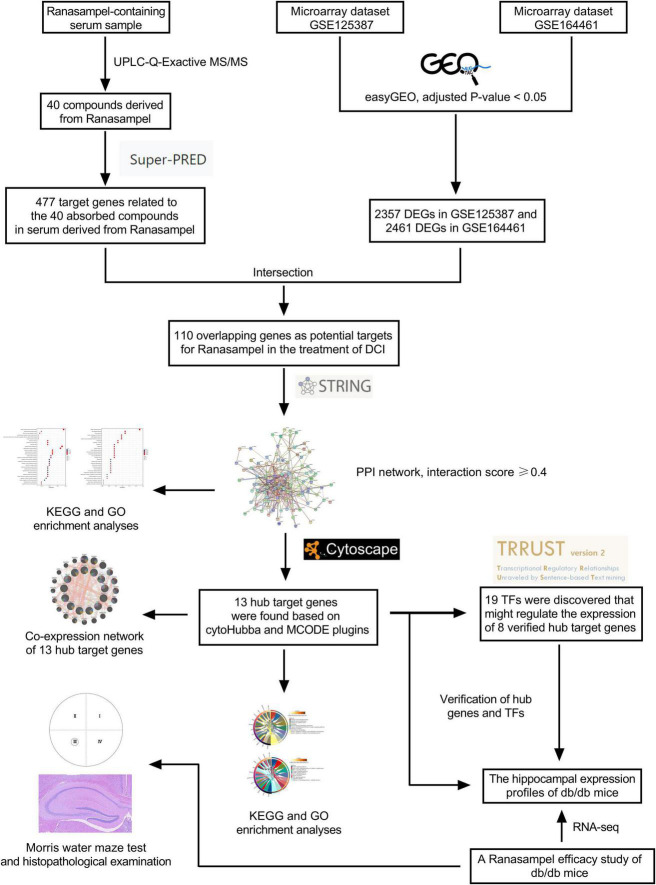
Flow chart of serum pharmacochemistry study and microarray data analysis of Ranasampel for diabetes cognitive impairment (DCI) treatment.

Nowadays, much remains unknown about the deeper mechanisms underlying the association between diabetes and AD, and there are no specific drugs to prevent and treat diabetes-related cognitive dysfunction. So, the use of the Tibetan medicinal formula Ranasampel may provide a new vision for the prevention and treatment strategy of diabetic cognitive impairment. Therefore, in this study, we firstly identified the blood-entering active components and performed target prediction of Ranasampel by serum pharmacochemistry. Then, two gene expression datasets (GSE125387 and GSE164461) from the Gene Expression Omnibus (GEO) database were obtained to screen the possible targets and corresponding mechanisms for the regulation of diabetic cognitive impairment by the serum active ingredients of Ranasampel through bioinformatics study. Finally, the relevant target genes were validated by a microarray dataset of db/db mice treated with Ranasampel, to explore the possible pharmacodynamic basis of Ranasampel and provide new ideas and insights for the pharmacology research related to the prevention and treatment of cognitive impairment in diabetes. The flowchart of this study was shown in Figure 1.

## 2 Materials and methods

### 2.1 Preparation of drug-containing serum

According to the *Chinese Pharmacopeia* (2015 version), the Tibetan medicinal formula Qishiwei Zhenzhu Pills (Ranasampel) consists of more than 70 traditional pharmaceutical ingredients, including *Margarita* (pearl), *Lignum Santali Albi* (sandalwood), *Lignum Dalbergiae Odoriferae* (rosewood), *Benzoinum* (benzoin), *Radix Glycyrrhizae* (liquorice root), *Lagotis Brachystachya* (Shortspike Lagotis), *Concretio Silicea Bambusae* (tabasheer), *Croci Stigma* (saffron), *Fructus Chebulae* (medicine terminalia fruit), *Moschus* (Musk), *Calculus Bovis* (bezoar), and *Cornu Saigae Tataricae* (antelope horn) (Schwabl and Vennos, 2015; Fu et al., 2020). The Ranasampel used in the study were purchased from the Ganlu Tibetan Medicine Co., Ltd., Tibet Autonomous Region, China (Lot No. 210500114).

The male C57BL/6 mice aged 10-week-old were purchased from the Beijing Vital River Laboratory Animal Technology Co., Ltd. [No. SYXK (Jing) 2016-0006, Beijing, China]. The animals were housed in a standard animal feeding room (room temperature: 22 ± 2°C; relative humidity: 50 ± 15%; light condition: 12 h:12 h light: dark cycle) and fed a standard rodent diet. The clinical dosage of Ranasampel for adults (standard weight 70 kg) is 1 g/day (Nie et al., 2021), therefore the dose for animal experiments was calculated according to the body surface area-based equivalent dose calculation method (Chatelut and Puisset, 2014) for humans and mice, and the gavage dose for mice was 0.125 g/kg/day.

This study was performed in agreement with the recommendations of the existing current animal welfare guidelines. The animal experimental protocols were approved by the Institutional Animal Care and Use Committee of the Beijing University of Chinese Medicine (No. BUCM-4-2022012105-1060). A total of 80 mice were assigned to 8 groups (*n* = 10), control group with no gavage, Ranasampel groups were administrated with Ranasampel in doses of 0.125 g/kg (group 1x); 0.25 g/kg (group 2x); 0.5 g/kg (group 4x); 0.75 g/kg (group 6x); 1.0 g/kg (group 8x); 1.25 g/kg (group 10x); and 1.5 g/kg (group 12x). The drug-containing serum of Ranasampel was prepared as previously reported (Ge et al., 2010; Du et al., 2020). Briefly, drug intervention lasted for 1 week, and fasting for 12 h after gavage on day 6. The abdominal aortic blood was taken under aseptic condition within 2 h after gavage on day 7, then centrifuged at 4°C 4,000 r/min for 10 mins to obtain Ranasampel drug-containing serum. Finally, serum samples were inactivated by water bath at 56°C for 30 mins and filtered through a 0.22 μm filter membrane, then stored at −80°C for the serum pharmacochemistry analysis.

### 2.2 Serum pharmacochemistry approach

The serum pharmacochemistry method was performed to identify the serum components of Ranasampel using Vanquish UHPLC System with Q-Exactive HF Mass Spectrometer (Thermo Fisher Scientific) (Wang et al., 2019), then the network pharmacological method was used to predict the targets of identified compounds in the drug-containing serum.

Chromatographic separation was conducted on a Zorbax Eclipse C18 column (1.8 μm × 2.1 × 100 mm, Agilent technologies, CA, United States). The column temperature was set at 30°C and the flow rate was 0.3 mL/min. The mobile phase consisted of deionized water with 0.1% formic acid (A) and pure acetonitrile (B). The gradient elution program was as follows: 0–2 min, 5% B; 2–6 min, 30% B; 6–7 min, 30% B; 7–12 min, 78% B; 12–14 min, 78% B; 14–17 min, 95% B; 17–20 min, 95% B; 20–21 min, 5% B; 21–25 min, 5% B.

The mass spectrometer analysis was performed using an ESI ion source in both positive and negative ion modes. The heating temperatures and capillary temperatures were 330°C and 335°C, respectively. The flow rates of the sheath gas, the auxiliary gas, and the sweep gas were 45 arb, 15 arb, and 1 arb, respectively. The electrospray voltage was 3.5 kV and the S-Lens RF Level was 55%. The LC-MS data were collected in the Full Scan/ddMS2 mode (m/z 100–1500; Top *N* = 10) and analyzed by Compound Discoverer 3.2. According to the processing parameters (ppm; mass tolerance; RT tolerance; and Area) and mass spectrometry information (mass spectrometry fragment patterns; chemical composition; chromatographic elution behavior; and corresponding reference alignments), the compounds were identified by using the Thermo mzCloud online database and the Thermo mzValut local database (Rohde et al., 2021; Wang et al., 2021). SMILES files of the compounds of Ranasampel drug-containing serum were obtained from PubChem^1^ (Kim et al., 2021) and ChemSpider^2^ (Little et al., 2012) databases, then the target genes were predicted through the Super-PRED^3^ (Zhou et al., 2019) database. The Cytoscape (version 3.7.2^4^, Boston, MA, United States) (Wu et al., 2020) was used to establish the “Active ingredients in drug-containing serum of Ranasampel − Predicted target genes” network.

### 2.3 Collation of datasets

The GEO database^5^, a public database created and maintained by the National Center for Biotechnology Information (NCBI), archives and freely distributes a large number of high-throughput gene expression and other functional genomics data submitted by research institutions worldwide (Clough and Barrett, 2016). “Alzheimer’s disease,” “diabetes mellitus,” and “cognitive dysfunction” were the search terms, while the test specimen included was mice. Two microarray datasets (GSE125387 and GSE164461) including RNA expression profiling were screened out. The GSE125387 dataset was performed on mice hippocampal tissue samples that differentiate between the db/db mice and db/m mice, while the GSE164461 dataset contained APPswe/PSEN1dE9 mice and wild-type mice. The platform used to analyze these data was the GPL23479 BGISEQ-500 (Mus musculus) (Zhang et al., 2022).

### 2.4 Identification of Ranasampel target genes

The easy Visualization and Inference Toolbox for Transcriptome Analysis (eVITTA^6^) (Cheng et al., 2021) is a web-based toolbox for transcriptome analysis, which provides an R package (edgeR and limma)-based online tool for analysis and exploration of studies published in NCBI GEO (easyGEO). Microarray data from the GSE125387 and GSE164461 datasets were submitted to the easyGEO tool^7^ to determine differentially expressed genes (DEGs) in the hippocampal tissue samples between the diseased group and the control group. The genes with adjusted *P*-value < 0.05 were considered as DEGs, and the probes without corresponding gene symbols were excluded. Then, the predicted target genes of Ranasampel and the DEGs of two datasets were overlapped by the online Venn diagram tool to obtain the potential therapeutic target genes of Ranasampel for diabetes cognitive impairment. The DEGs and Ranasampel target genes were visualized using volcano plots and hierarchical clustering heat maps (R package: ggplot2 and pheatmap).

### 2.5 Protein–protein interaction network analysis

The Search Tool for the Retrieval of Interacting Genes (version 11.0) (STRING^8^) (Szklarczyk et al., 2019) was used to construct a protein–protein interaction (PPI) network of potential target genes of Ranasampel with complex regulatory relationships. The interaction score ≥ 0.4 were considered statistically significant. The Gene ontology (GO) function and Kyoto Encyclopedia of Genes and Genomes (KEGG) pathway analysis of the Ranasampel target genes was performed with the R package (clusterProfiler), and the enrichment analysis results with adjusted *P*-value < 0.05 were considered significant. Then, Cytoscape was used to visualize the network, and the molecular complex detection technology (MCODE) plugin of the software was used to analyze functional sub-modules. The selection criteria were set as follows: degree cutoff = 2, node score cutoff = 0.2, K-core = 2 and max depth = 100.

### 2.6 Screening and analysis of hub genes

The target genes in the PPI network were identified with the cytoHubba plugin of Cytoscape. Here, the common algorithms (MCC, MNC, EPC, Degree, Closeness, Stress, and Radiality) were used to explore the key genes (Su et al., 2021). Subsequently, the GO and KEGG enrichment analysis was used to identify the biological function of modules containing these key genes, while the key genes in the modules were defined as hub genes. Then, a co-expression network of these hub genes was constructed based on the GeneMANIA^9^ (Warde-Farley et al., 2010), which is a large set of functional association databases to find protein and genetic interactions, pathways, co-expression, co-localization, and protein domain similarity of gene sets.

### 2.7 RNA microarray verification of hub genes expression

A Ranasampel efficacy study of db/db mice was used to validate hub genes. Eight-week-old male db/db and db/m mice were purchased from Changzhou Cave Laboratory Animal Co., Ltd. (No. SCXK (Su) 2016-0010, Changzhou, China). Mice were divided into 3 groups (*n* = 11 for each group): (1) control group (db/m), (2) diabetes cognitive impairment (DCI) group (db/db), and (3) Ranasampel group (db/db + Ranasampel). All mice were housed in the Beijing University of Chinese Medicine animal feeding room under standard conditions with a temperature of 22 ± 2°C, relative humidity at 50 ± 15%, and 12 h:12 h light: dark cycle. Mice were fed regular chow and pure water. Control group and DCI group mice with no gavage, while Ranasampel group mice were daily administrated with Ranasampel in doses of 1.25 g/kg (10x the dose of Ranasampel based on serum pharmacochemistry study) for 4 weeks starting at 10 weeks of age. Then, 8 mice from each group were sacrificed to collect hippocampal tissues for RNA microarray analysis.

The RNA concentration and purity of hippocampal tissue samples were measured using NanoDrop 2000 (Thermo Fisher Scientific, DE, United States). RNA integrity was assessed using the RNA Nano 6000 Assay Kit of the Agilent Bioanalyzer 2100 system (Agilent Technologies, CA, United States). A total amount of 1 μg RNA per sample was used as input material for the RNA sample preparations. Sequencing libraries were generated using NEBNext UltraTM RNA Library Prep Kit for Illumina (NEB, United States) and index codes were added to attribute sequences to each sample (Tvedte et al., 2021). The clustering of the index-coded samples was performed on a cBot Cluster Generation System with TruSeq PE Cluster Kit v4-cBot-HS (Illumina) (Lynch et al., 2015). Then, the library preparations were sequenced on an Illumina platform and paired-end reads were generated. The raw reads were further processed with BMKCloud^10^ online platform, which is a bioinformatic pipeline tool, and the raw data (raw reads) of fastq format were processed through in-house perl scripts. At last, the quantification of gene expression was estimated by fragments per kilobase of transcript per million fragments mapped (FPKM), and the FPKM values were used to evaluate the expression levels of hub genes.

### 2.8 Behavior and histopathological assessment

The spatial learning and memory behaviors of the DCI mice in the Ranasampel efficacy study were measured using Morris water-maze test. The protocol was conducted and modified as previously described (Jiang et al., 2021). In brief, the water maze apparatus (XRXM101, Shanghai Xinruan Information Technology Co., Ltd., China) consists of a circular pool (diameter, 150 cm; height, 35 cm) and a high-definition video recorder connected with an automated tracking system. The pool area was equally divided into four quadrants (I, II, III, and IV). The hidden platform trials were conducted for 5 consecutive days (day 1–day 5) with a visible platform placed at quadrant III. The poll was filled with opaque water (temperature, 24 ± 1°C) which helped to hide the submerged part of the platform. On day 6, the platform was removed and a probe trial was conducted. The latency to the platform and the time spent in the target quadrant of mice in 60 s were recorded, and the data were automatically analyzed by SuperMaze software (Shanghai Xinruan Information Technology Co., Ltd, China).

The whole brain tissues of 3 mice in each group were fixed in a 4% PFA solution to prepare brain sections which included hippocampal cornu ammonis 1 (CA1), cornu ammonis 3 (CA3), and dentate gyrus (DG) regions based on the stereotaxic atlases of the mouse brain (Konsman, 2003). The sections were subjected to hematoxylin-eosin (H&E) staining to observe the hippocampal neurocytes and inflammatory cell infiltration. A four-point severity scale (0, normal; 1, mild; 2, moderate; 3, severe) was used to score the H&E stained sections by two experienced pathologists in a blinded manner (Yan et al., 2021).

### 2.9 Prediction and validation of transcription factors

The Transcriptional Regulatory Relationships Unraveled by Sentence-based Text mining (TRRUST^11^) (Han et al., 2018) database was used to obtain transcription factors (TFs) that regulate the validated hub genes, then the expressions of these TFs were verified in the microarray data. The transcriptional regulatory network of hub genes and TFs was visualized by Cytoscape software.

### 2.10 Statistical analysis

Except for the RNA sequencing data, the visualized data were expressed as means ± SEM, and the comparison between groups was performed by one-way ANOVA with T-test when equal variances were assumed. When the data had a normal distribution but the variances were not homogeneous, then Dunnett’s T3 test was used. *P*-value < 0.05 was considered statistically significant and the statistics were visualized by R package (ggplot2).

## 3 Results

### 3.1 Serum pharmacochemistry analysis

**FIGURE 2:**
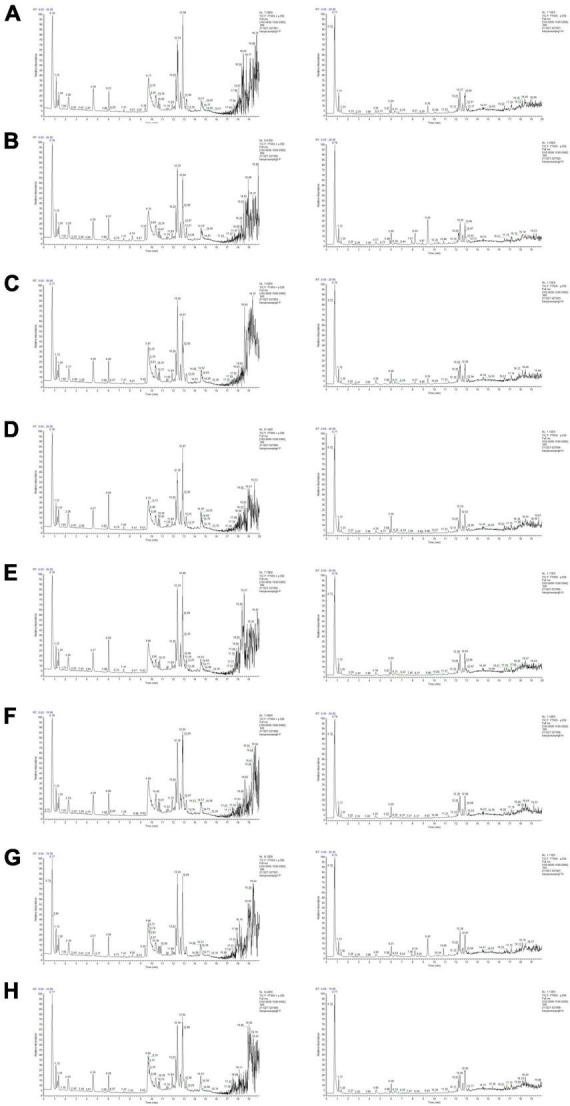
Total ion chromatographs of drug-containing serum samples from mice with oral administration of Ranasampel. **(A)** Serum samples of control group; **(B)** Serum samples of 1x dose group (0.125 g/kg); **(C)** Serum samples of 2x dose group (0.25 g/kg); **(D)** Serum samples of 4x dose group (0.5 g/kg); **(E)** Serum samples of 6x dose group (0.75 g/kg); **(F)** Serum samples of 8x dose group (1.0 g/kg); **(G)** Serum samples of 10x dose group (1.25 g/kg); **(H)** Serum samples of 12x dose group (1.5 g/kg).

After optimizing the chromatogram and mass spectrometric conditions, the UPLC-Q-Exactive MS/MS method was used to identify the serum compounds of Ranasampel. As shown in Figure 2, drug-containing serum samples from mice administered with Ranasampel were analyzed using both positive and negative ion modes. A total of 40 compounds derived from Ranasampel were identified according to the mzCloud and mzValut databases. These compounds were summarized in Table 1, which included organic acids, steroids, volatile oils, terpenoids, etc. In addition, the serum compounds of mice in the 10x dose group (1.25 g/kg) had a more comprehensive composition than the rest of the dose groups. However, the prototype components and metabolites in serum were not identified because of the complexity of Ranasampel compound metabolites.

**TABLE 1:** UPLC-Q-Exactive MS/MS method was used to identify the active components in Ranasampel-containing serum.

No.	TR/min	Name	Formula	Error/ppm	[M-H]^–^/ [M + H]^+^	Class
1	0.717	L(+)-Ornithine	C5H12N2O2	0.65	/132.08988	Carboxylic acids and derivatives
2	0.719	DL-Lysine	C6H14N2O2	0.15	/146.10553	Carboxylic acids and derivatives
3	0.738	(2E)-2,5-Dichloro-4-oxo-2-hexenedioic acid	C6H4Cl2O5	2.41	/225.94358	Others
4	0.809	Taurine	C2H7NO3S	0.76	/125.01466	Organic sulfonic acids and derivatives
5	0.914	Benzoic acid	C7H6O2	1.41	/122.03678	Benzene and substituted derivatives
6	0.918	L-Tyrosine	C9H11NO3	0.33	/181.07389	Carboxylic acids and derivatives
7	1.147	L-Methionine sulfoxide	C5H11NO3S	0.17	/165.04596	Carboxylic acids and derivatives
8	2.291	p-Coumaraldehyde	C9H8O2	0.53	/148.05243	Cinnamaldehydes
9	2.865	D-Pantothenic acid hemicalcium salt	C9H17NO5	0.27	-/219.11067	Others
10	5.566	Hippuric acid	C9H9NO3	–0.02	/179.05824	Benzene and substituted derivatives
11	6.007	N-[(9H-Fluoren-9-ylmethoxy)carbonyl]-L-tryptophyl-L-valyl-L-phenylalanyl-L-phenylalanine	C49H49N5O7	–0.27	-/819.3632	Others
12	6.255	N-Acetyl-L-leucine	C8H15NO3	0.28	/173.10519	Carboxylic acids and derivatives
13	6.596	4-Coumaric acid	C9H8O3	0.3	/164.04734	Cinnamic acids and derivatives
14	6.755	N-acetylphenylalanine	C11H13NO3	–0.07	-/207.08954	Carboxylic acids and derivatives
15	8.206	1-[2-(Dimethylamino)-6-{[3-(5-methyl-4-phenyl-1H-imidazol-1-yl) propyl]sulfanylphenyl]-3-pentylurea	C27H37N5OS	–2.4	/479.27188	Others
16	9.004	ginkgolide B	C20H24O10	3.2	-/424.13695	Prenol lipids
17	9.46	Mebeverine	C25H35NO5	–0.08	/429.25152	Benzene and substituted derivatives
18	9.993	Glycocholic acid	C26H43NO6	2.38	-/465.30904	Steroids and steroid derivatives
19	10.385	Taurochenodeoxycholic acid	C26H45NO6S	2.64	-/499.29676	Steroids and steroid derivatives
20	10.858	21-Desacetyl deflazacort	C23H29NO5	–0.22	/399.20457	Steroids and steroid derivatives
21	10.865	7-ketodeoxycholic acid	C24H38O5	2.98	-/406.27192	Steroids and steroid derivatives
22	10.866	5b-CHOLANIC ACID-3a,12a-DIOL-7-ONE	C24H38O5	–0.38	/406.27192	Steroids and steroid derivatives
23	11.068	Docosahexaenoic acid ethyl ester	C24H36O2	–0.79	/356.27153	Fatty Acyls
24	12.102	LPE 18:2	C23H44NO7P	1.94	-/477.28554	Glycerophospholipids
25	12.127	Phosphatidylethanolamine lyso 22:6	C27H44NO7P	2.11	-/525.28554	Glycerophospholipids
26	12.682	Palmitoleic acid	C16H30O2	–1.02	/254.22458	Fatty Acyls
27	14.243	1-Stearoyl-sn-glycero-3-phosphocholine	C26H54NO7P	–0.16	/523.36379	Glycerophospholipids
28	14.434	Dodecyl sulfate	C12H26O4S	1.66	-/266.15518	Organic sulfuric acids and derivatives
29	16.399	Hexadecanamide	C16H33NO	–0.56	/255.25621	Fatty Acyls
30	17.964	α-Methylhistamine	C6H11N3	1.55	/125.0953	Others
31	18.282	2-Amino-4-methylpyrimidine	C5H7N3	1.28	/109.064	Diazines
32	18.367	2-Aminobenzimidazole	C7H7N3	–1.18	/133.064	Benzimidazoles
33	18.449	Ethyl palmitoleate	C18H34O2	–0.45	/282.25588	Fatty Acyls
34	18.755	11(Z),14(Z)-Eicosadienoic acid	C20H36O2	1.98	-/308.27153	Fatty Acyls
35	18.872	Hydralazine	C8H8N4	–0.5	/160.0749	Diazanaphthalenes
36	18.902	Stearamide	C18H37NO	–1.32	/283.28751	Carboximidic acids and derivatives
37	19.001	1-Stearoylglycerol	C21H42O4	–1.16	/358.30831	Glycerolipids
38	19.014	Vinylpyrazine	C6H6N2	–0.38	/106.0531	Diazines
39	19.195	N1-[2-(4-Pyridyl)ethyl]ethanimidamide	C9H13N3	–1.4	/163.11095	Pyridines and derivatives
40	19.918	cis,cis-Muconic acid	C6H6O4	4.74	-/142.02661	Fatty Acyls

**FIGURE 3:**
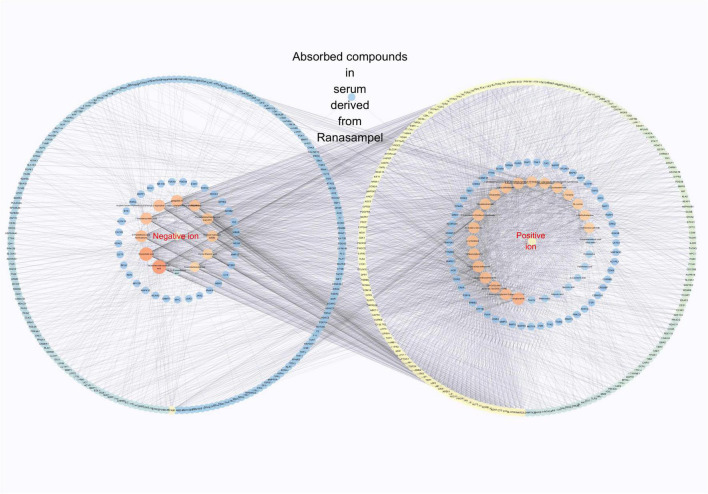
Active ingredients in drug-containing serum of Ranasampel – predicted target genes” network. These 40 absorbed components in serum derived from Ranasampel could regulate 477 potential target genes.

Forty absorbed components were considered to be the potential active compounds in serum containing Ranasampel. These absorbed components were then used to predict the potential targets of Ranasampel. By searching the Super-PRED database, a total of 477 target genes related to the 40 absorbed compounds in serum derived from Ranasampel were predicted. As shown in Figure 3, the “active compounds in the drug-containing serum of Ranasampel − predicted target genes” network were constructed using Cytoscape software.

### 3.2 Analysis of the Ranasampel target genes

**FIGURE 4:**
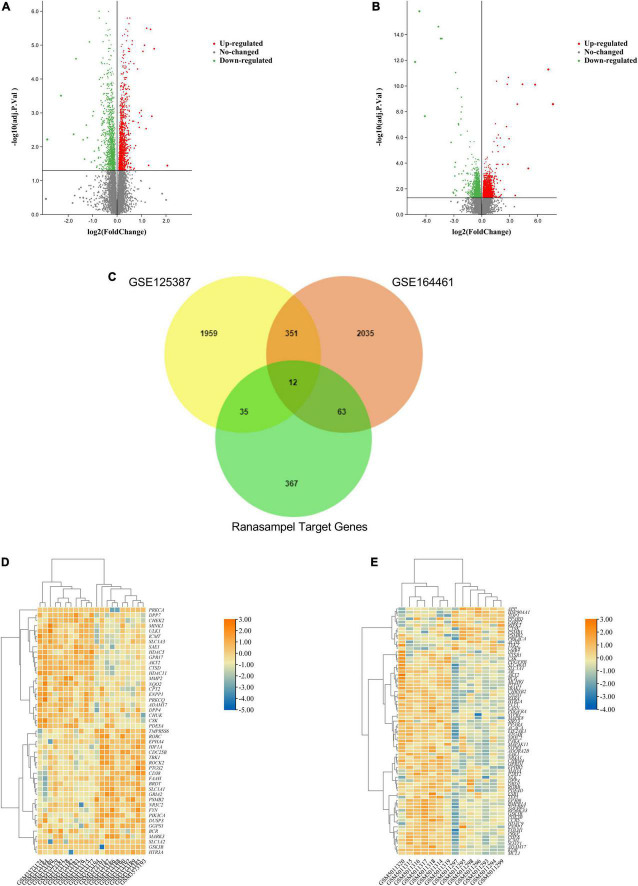
Differentially expressed genes of RNA microarray results and potential targets of Ranasampel for DCI. **(A)** The volcano map of GSE125387; **(B)** The volcano map of GSE164461. Red dots represent upregulated genes, green dots represent downregulated genes, *adjust P* < 0.05. **(C)** Venn diagram showed that an overlap of 47 potential target genes in GSE125387, 75 potential target genes in GSE164461, and 110 potential target genes for Ranasampel in the treatment of DCI; **(D)** The heat map of Ranasampel target genes in GSE125387; **(E)** The heat map of Ranasampel target genes in GSE164461.

After standardizing the microarray results, DEGs (2357 in GSE125387 and 2461 in GSE164461, Supplementary Tables 1, 2) were identified (Figures 4A, B). Then, the potential target genes of Ranasampel and these DEGs were taken the intersection. As shown in the Venn diagram (Figure 4C), 110 overlapping genes were identified, which were considered as potential targets for Ranasampel in the treatment of DCI. The heat maps of Ranasampel target genes in the GSE125387 and GSE164461 were also displayed (Figures 4D, E).

**FIGURE 5:**
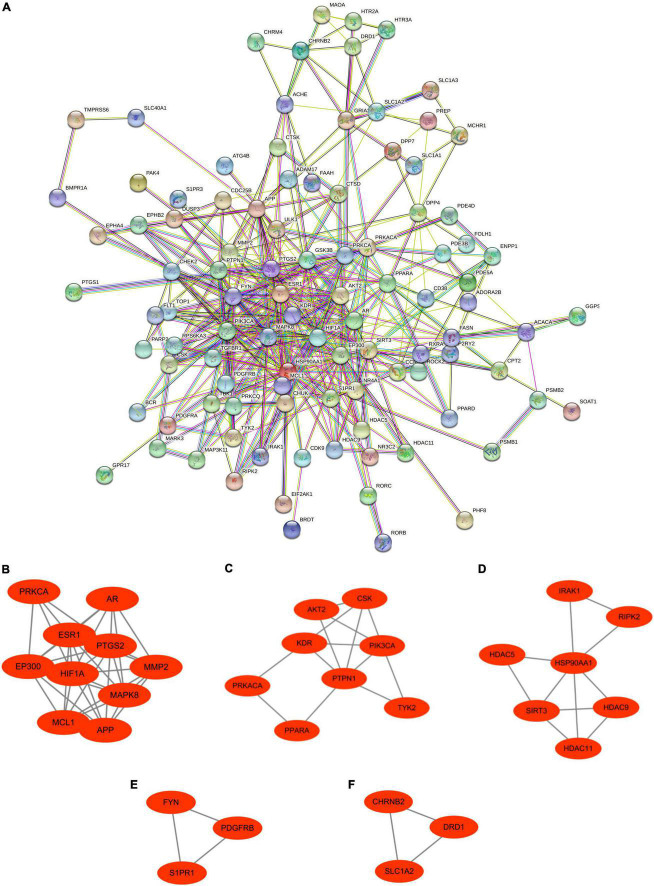
Protein–protein interaction network and significant gene modules of Ranasampel target genes. **(A)** The PPI network contained 101 nodes and 402 edges; **(B–F)** Five significant gene clustering modules contained 31 nodes and 67 edges.

These Ranasampel target genes with interaction scores greater than 0.4 were used to establish the PPI network in the STRING website, which contained 101 nodes and 402 edges (Figure 5A). Meanwhile, the PPI network file was maintained from STRING for module analysis by using Cytoscape. Five crucial sub-modules were screened through the MCODE plugin of Cytoscape, including 31 nodes and 67 edges (Figures 5B-F).

**FIGURE 6:**
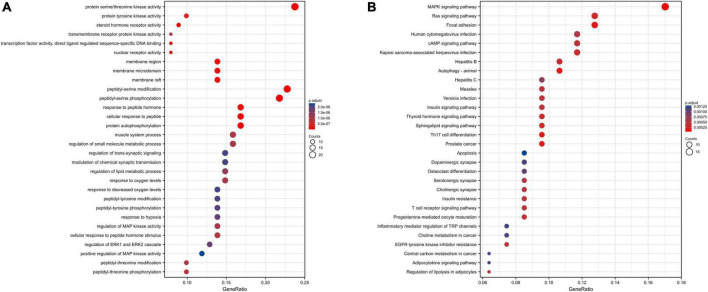
Functional enrichment analysis of interact genes in the PPI network. **(A)** Top 30 enriched GO terms; **(B)** Top 30 enriched KEGG pathways. The abscissa represented the frequency of the genes involved in total genes of this GO term, and the ordinate indicated the functional description of the enriched GO term. The size of the circle represented the number of genes enriched to this function, and the chromatograms from red to blue represented the adjusted *P*-value.

Furthermore, to analyze the biological functions and signaling pathways involved in the 101 interact genes, GO and KEGG enrichment analyses were performed. GO analysis results exhibited that these genes were mainly enriched in protein serine/threonine kinase activity (*adjust P* = 1.15195E-14), response to oxygen levels (*adjust P* = 1.04485E-06), peptidyl-serine phosphorylation (*adjust P* = 7.08448E-16), protein autophosphorylation (*adjust P* = 9.4354E-12), and regulation of ERK1 and ERK2 cascade (*adjust P* = 1.82485E-06) (Figure 6A). These functional terms are relevant to systemic inflammation and neuroinflammation, which is critical in metabolic and central diseases. KEGG analysis results showed that genes to be mainly involved in the MAPK signaling pathway (*adjust P* = 5.00047E-05), Cholinergic synapse (*adjust P* = 0.000533567), Insulin resistance (*adjust P* = 0.000446102), cAMP signaling pathway (*adjust P* = 0.000475452), and Th17 cell differentiation (*adjust P* = 0.000171201) (Figure 6B), indicated that these pathways might be closely related to the Ranasampel efficacy.

### 3.3 Identification and analysis of hub genes

**FIGURE 7:**
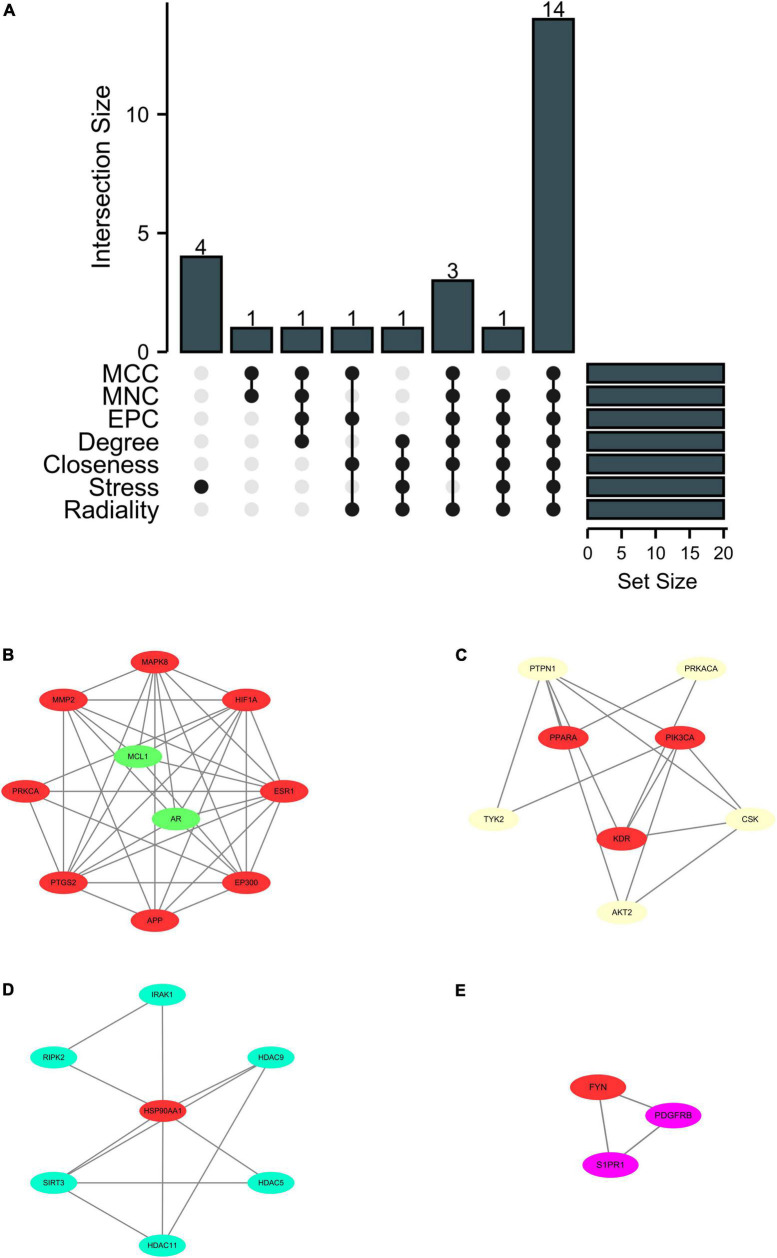
UpSet plot and modules overlapped with hub genes. **(A)** The UpSet plot represented 14 hub genes obtained from the cytoHubba plugin; **(B–E)** Four significant gene clustering modules from the PPI network overlapped 13 hub target genes. Red nodes represented the hub target genes in modules.

To explore the key target genes of Ranasampel, the top 20 hub genes of the PPI network were calculated through the 7 algorithms of the cytoHubba plugin (Supplementary Table 3). The UpSet plot was used to demonstrate the common genes obtained by the 7 algorithms (Figure 7A). A total of 14 hub genes were discovered, including APP, EP300, ESR1, FYN, GSK3B, HIF1A, HSP90AA1, KDR, MAPK8, MMP2, PIK3CA, PPARA, PRKCA, and PTGS2. Then, after overlapping these hub genes with sub-modules of the PPI network, 13 hub target genes were found in 4 modules (Figures 7B-E).

**FIGURE 8:**
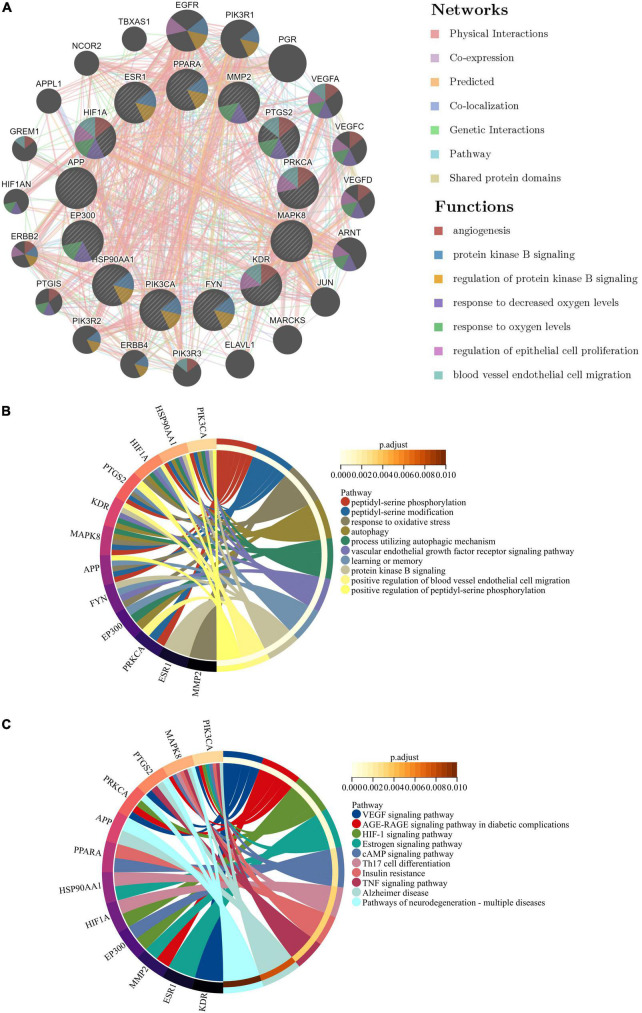
Co-expression network and enrichment analysis of module overlapped hub target genes. **(A)** Thirteen hub target genes and their co-expression genes were analyzed via GeneMANIA; **(B, C)** GO and KEGG analysis of 13 hub target genes. The outermost circle on the right represented GO and KEGG terms, and the inner circle on the left represented the significant adjust *P*-value of enrichment results.

Based on the GeneMANIA database, the co-expression network and related functions of these 13 hub target genes were analyzed. Among 13 hub genes with interacting proteins, it was found that 77.64% had physical interactions, 8.01% exerted co-localization, 5.37% of the predicted rate, 3.63% displayed co-localization, 2.87% exhibited genetic interactions, 1.88% existed pathway and 0.6% shared protein domains (Figure 8A). To study further the 13 hub target genes, GO and KEGG enrichment analyses were carried out. GO analysis showed that 13 hub target genes were mainly involved in vascular endothelial growth factor receptor signaling pathway (*adjust P* = 6.60E-06), peptidyl-serine phosphorylation (*adjust P* = 2.05E-05), response to oxidative stress (*adjust P* = 5.00047E-05), autophagy (*adjust P* = 1.12E-04) and learning or memory (*adjust P* = 1.12E-04) (Figure 8B). The GO terms of results emphasized that the efficacy of Ranasampel might be relevant to the mechanisms of vascular endothelial growth, oxidative stress, inflammation, autophagy, and learning or memory. Meanwhile, KEGG analysis showed that these genes were mainly involved in VEGF signaling pathway (*adjust P* = 7.00E-05), AGE-RAGE signaling pathway in diabetic complications (*adjust P* = 2.32E-04), HIF-1 signaling pathway (*adjust P* = 2.97E-04), Insulin resistance (*adjust P* = 3.11E-03) and Alzheimer disease (*adjust P* = 7.36E-03) (Figure 8C).

### 3.4 Ranasampel alleviated cognitive impairment in db/db mice

**FIGURE 9:**
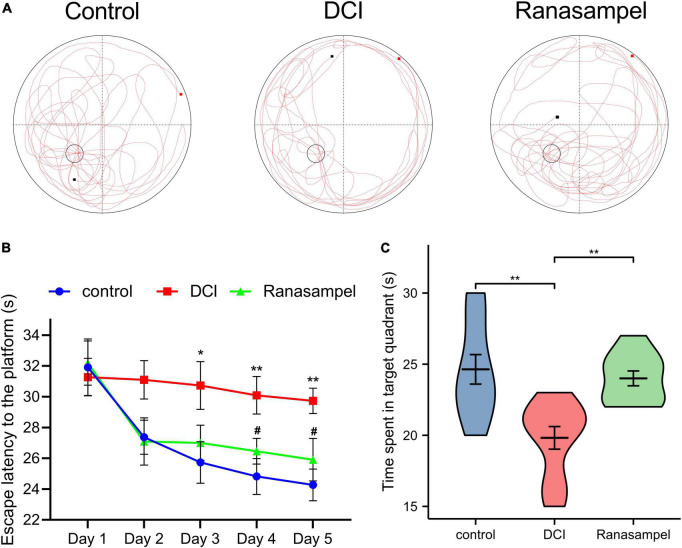
The Morris water-maze results of the Ranasampel efficacy study in db/db mice. **(A)** The representative moving trails in the Morris water-maze test of mice in each group; **(B)** The escape latency to the platform during the 5-day hidden platform trials (*n* = 11; *, DCI group compared with the control group; #, Ranasampel group compared with DCI group); **(C)** The results of the time spent in the target quadrant in the probe trial (*n* = 11). Data were expressed as means ± SEM, *P < 0.05; **P < 0.01; ^#^*P* < 0.05.

The Morris water-maze test was performed to assess the effects of Ranasampel on cognitive impairment in db/db diabetic mice. As shown in Figure 9, the 5-day hidden platform trials reflected that Ranasampel improved learning and memory deficits of db/db mice (*P* < 0.05). Furthermore, the probe trial on day 6 observed the time spent in the target quadrant indicating Ranasampel increased the spatial memory of db/db mice (*P* < 0.01).

**FIGURE 10:**
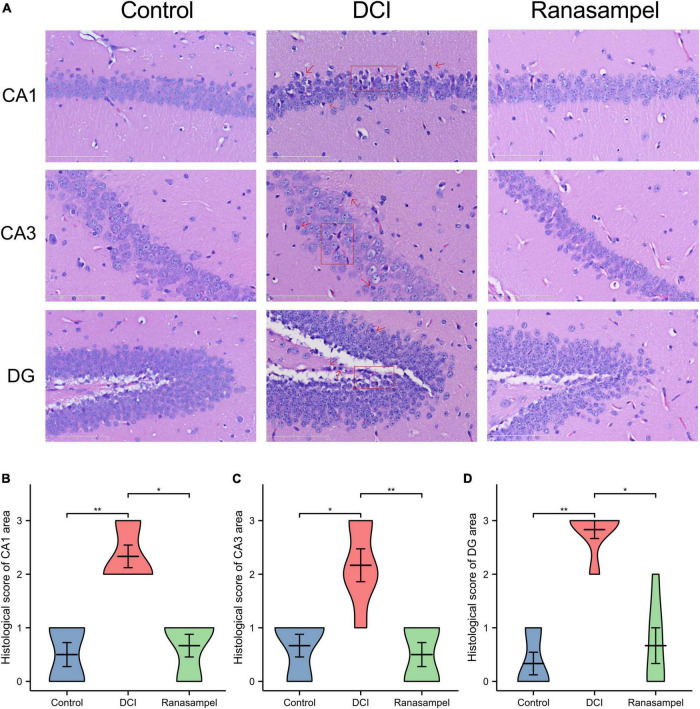
The histological changes in CA1, CA3, and DG regions of the mouse hippocampus. **(A)** Representative micrographs of H&E staining (scale bar = 200 μm, 400 × magnification) in the CA1 and CA3 regions (red arrows: hyperchromatic nuclei; red boxes: tissue cavitation and neuronal loss); **(B)** The histological scores in the CA1 region (*n* = 3); **(C)** The histological scores in the CA3 region (*n* = 3); **(D)** The histological scores in the DG region (*n* = 3). Data were expressed as means ± SEM, *P < 0.05; **P < 0.01.

Next, H&E staining was performed on the histopathological changes in mice hippocampal CA1, CA3, and DG regions to evaluate the neuronal damage and neuroinflammation. As exhibited in Figure 10A, the obvious neuronal loss, tissue cavitation, and abnormal shapes with hyperchromatic nuclei were found in the DCI group, while Ranasampel notably mitigated these histopathological changes, as determined according to the morphology and histological scores of the mouse hippocampus (*P* < 0.05 or *P* < 0.01) (Figures 10B-D).

### 3.5 Verification of hub genes expression

**FIGURE 11:**
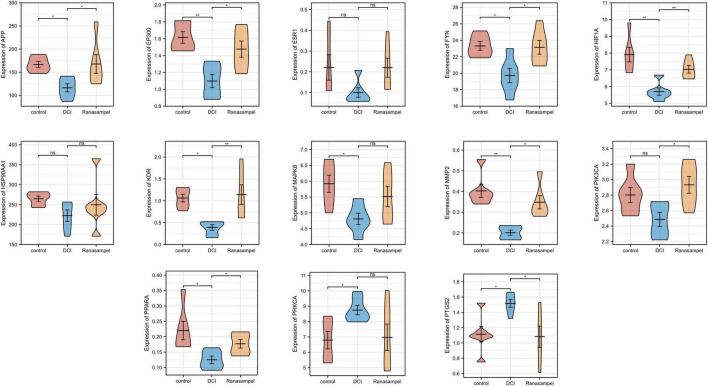
The expression levels of hub target genes in the hippocampal expression profiles of db/db mice after Ranasampel treatment. Data were expressed as means ± SEM, *P < 0.05; **P < 0.01. ns, no significance.

The hippocampal expression profiles of db/db mice after Ranasampel treatment (Supplementary Table 4) were used to verify the reliability of 13 hub target gene expression levels. The results showed that compared with the DCI group, the expressions of APP, EP300, FYN, HIF1A, KDR, MMP2, PIK3CA, and PPARA were significantly up-regulated (*P* < 0.05 or *P* < 0.01), while the expression of PTGS2 was significantly down-regulated (*P* < 0.05) (Figure 11). However, the expression levels of ESR1, HSP90AA1, MAPK8, and PPKCA had no significant changes in this microarray dataset.

### 3.6 Prediction and validation of TFs

**FIGURE 12:**
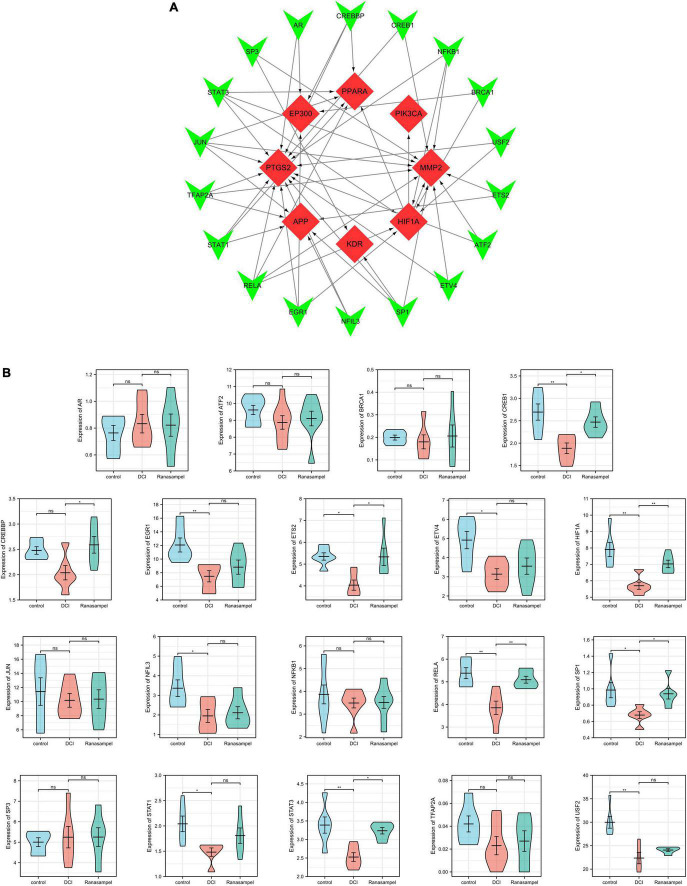
Transcription factors (TFs) regulatory network and validation of TFs. **(A)** The “TFs – verified hub target genes” network, TFs were marked in green, and the verified hub target genes were marked in red; **(B)** The expressions of TFs in the hippocampal expression profiles of db/db mice after Ranasampel treatment. Data were expressed as means ± SEM, *P < 0.05; **P < 0.01. ns, no significance.

According to the TRRUST database, 19 TFs were discovered that might regulate the expression of 8 verified hub target genes (No TFs of FYN were found in the database; HIF1A was both the hub target gene and transcription factor) (Figure 12A and Table 2). Further validation, the expressions of CREB1, CREBBP, ETS2, HIF1A, RELA, SP1, and STAT3 were highly expressed in the dataset (*P* < 0.05 or *P* < 0.01) (Figure 12B). They synergistically participated in the regulation of 7 hub target genes (APP, EP300, HIF1A, MMP2, PIK3CA, PPARA, and PTGS2).

**TABLE 2:** Key transcription factors of 8 verified hub target genes.

Key TFs	Description	*P*-value	List of overlapped genes
HIF1A	Hypoxia inducible factor 1, alpha subunit (basic helix-loop-helix transcription factor)	4.31E-08	MMP2, PIK3CA, PPARA, HIF1A
CREBBP	CREB binding protein	1.72E-07	EP300, PTGS2, PPARA
STAT3	Signal transducer and activator of transcription 3 (acute-phase response factor)	3.76E-07	PTGS2, MMP2, PPARA, HIF1A
JUN	Jun proto-oncogene	4.56E-07	MMP2, APP, PTGS2, PPARA
TFAP2A	Transcription factor AP-2 alpha (activating enhancer binding protein 2 alpha)	4.22E-06	MMP2, PTGS2, APP
STAT1	Signal transducer and activator of transcription 1, 91 kDa	7.01E-06	PPARA, APP, PTGS2
RELA	v-rel reticuloendotheliosis viral oncogene homolog A (avian)	7.50E-06	PPARA, MMP2, PTGS2, HIF1A
EGR1	Early growth response 1	8.07E-06	EP300, HIF1A, PTGS2
NFIL3	Nuclear factor, interleukin 3 regulated	9.08E-06	APP, PTGS2
SP1	Sp1 transcription factor	4.40E-05	MMP2, PTGS2, APP, KDR
ETV4	ets variant 4	4.65E-05	PTGS2, MMP2
ATF2	Activating transcription factor 2	9.96E-05	PTGS2, MMP2
ETS2	v-ets erythroblastosis virus E26 oncogene homolog 2 (avian)	9.96E-05	APP, MMP2
USF2	Upstream transcription factor 2, c-fos interacting	0.000216	PTGS2, HIF1A
BRCA1	Breast cancer 1, early onset	0.000318	EP300, HIF1A
NFKB1	Nuclear factor of kappa light polypeptide gene enhancer in B-cells 1	0.00032	MMP2, PTGS2, HIF1A
CREB1	cAMP responsive element binding protein 1	0.000793	MMP2, PTGS2
AR	Androgen receptor	0.000846	EP300, PTGS2
SP3	Sp3 transcription factor	0.00125	KDR, MMP2

## 4 Discussion

Clinical and epidemiological data suggest that diabetes is associated with less severe cognitive dysfunction and that people with diabetes are at high risk for cognitive dysfunction (Koekkoek et al., 2015; Biessels and Despa, 2018). According to statistics, 20% to 30% of people with diabetes will develop mild cognitive impairment and 17.3% will develop dementia (Alzheimer’s disease and vascular dementia), of which 20% of Type 2 diabetic patients over 60 years of age may develop dementia, with a significantly higher risk than non-diabetics (Albai et al., 2019; Kim et al., 2019). Therefore, given the increasing incidence of diabetes, the aging population and the impact of cognitive impairment on affected individuals and society as a whole, preventive treatment of diabetes is necessary (Zhang et al., 2018). However, while the evolving scientific literature does provide important clues, the understanding of potential therapeutic targets and underlying mechanisms of diabetes cognitive impairment (DCI) remains incomplete.

In recent years, the role of traditional Chinese medicine applied in the prevention and treatment of DCI has attracted much attention, whether it is classical prescriptions, traditional Chinese patent medicines, and simple preparations, or individual herbs, the active ingredients of which have unique advantages and efficacy, which deserve in-depth study (Seto et al., 2015). The efficacy of Ranasampel in improving vascular circulation, learning and memory functions, and neuroprotection is well documented for various cardiovascular and neurological diseases (Zhao et al., 2022). It is found that Ranasampel could reduce cerebral ischemia-reperfusion injury by inhibiting neuroinflammation and regulating gut microbiota in the rat cerebral ischemia model (Fu et al., 2021; Song et al., 2021). Also, Ranasampel may protect the neurons and glial cells by adjusting BBB function as well as lipid, fatty acid, and energy metabolism (Xu et al., 2020). Therefore, whether Ranasampel could be a complementary alternative medicine for the prevention and treatment of DCI also needed to be studied and demonstrated. In this study, we investigated the possible targets of Ranasampel to alleviate DCI and the related mechanisms by using serum medicinal chemistry and bioinformatics in this study, then verified these findings in a hippocampal expression profile of db/db mice treated with Ranasampel.

Traditional Chinese drugs are mostly administered orally and enter the bloodstream through the metabolism of various enzymes and microorganisms in the process of gastrointestinal and hepatic-intestinal circulation, which has multi-component, multi-pathway, and multi-target therapeutic characteristics, leading to complex and numerous components. This also indicates that the natural drugs do not only exert their therapeutic effect through direct action but may produce a combined effect through the integration of the natural drug ingredients, the metabolites of the natural ingredient and the endogenous physiologically active substances produced by the body as a result (Li et al., 2016). Serum pharmacochemistry is based on the serum after oral administration, considers blood components and their metabolites as the active ingredients for the action of a specific drug (Chen et al., 2022). Only by analyzing blood components can we avoid the blindness of *in vitro* chemical composition studies and the one-sidedness of taking individual components for pharmacokinetic studies, and be able to clarify the basis and mechanism of action of drugs in a more scientific manner.

In the present study, a total of 40 absorbed components were found in the Ranasampel-containing serum, which had 477 predicted target genes. The extensive overlap of these target genes across the components, suggesting that the therapeutic effects of Ranasampel are inextricably linked to the synergistic effects among the components. After that, we searched the GEO datasets and obtained hippocampal DEGs for diabetes and AD. Comparison of these DEGs with predicted targets identified 110 overlapping genes, predicting that Ranasampel might modulate these target genes and thereby alleviate DCI. The results of PPI network analysis acquired 101 targets of Ranasampel acting on DCI, of which 14 were hub genes. Then, 13 hub genes were cluster in modules of the PPI network, including APP, EP300, ESR1, FYN, GSK3B, HIF1A, HSP90AA1, KDR, MAPK8, MMP2, PIK3CA, PPARA, PRKCA, and PTGS2. The GO enrichment analysis revealed that these genes were significantly enriched in the vascular endothelial growth factor receptor (VEGF) signaling pathway, response to oxidative stress, positive regulation of blood vessel endothelial cell migration, autophagy, learning or memory, cognition, and positive regulation of peptidyl-serine phosphorylation. The KEGG results indicated that the therapeutic effect of Ranasampel in DCI mainly involved inflammatory and immune, diabetic complications and neurodegeneration pathways.

Insulin acts on tissues throughout the body to maintain plasma glucose within the physiological range by promoting glucose uptake and inhibiting glucose production and release by the liver (Wortham and Sander, 2016). Meanwhile, insulin also functions as an anabolic hormone that promotes fatty acid and amino acid uptake, energy storage, and cell growth (Saltiel, 2021). In addition, insulin also affects brain bioenergetics, enhances synaptic viability and dendritic spine formation, and increases neurotransmitter turnover. It also regulates vascular function through effects on vascular reactivity, lipid metabolism, and inflammation, and abnormal insulin regulation may lead to neurodegeneration (Kellar and Craft, 2020). Diabetes is characterized by elevated blood glucose levels caused by insufficient insulin production or insufficient insulin activity (Blüher, 2013). Among which, type 2 diabetes mellitus (T2DM) is caused by the inability of β cells to produce enough insulin to overcome systemic insulin resistance and is usually associated with obesity, inactivity, and aging (Van Dyken and Lacoste, 2018). Therefore, insulin resistance is a major pathogenic link in the development of T2DM and is emerging as a potentially important feature of Alzheimer’s disease and related dementias (Arnold et al., 2018). Most insulin in the brain is derived from circulating pancreatic insulin, which enters the brain mainly through selective, saturable transport across the capillary endothelium of the blood-brain barrier (BBB) (Unger and Betz, 1998; Banks et al., 2012). Insulin resistance prevents the normal intake and utilization of blood glucose, leading to loss of effective glucose control and causing hyperglycemia and T2DM. Hyperglycemia decreases BBB permeability to insulin, resulting in lower brain insulin levels and lower insulin-promoted neural and glial cell activity (Heni et al., 2014). On the other hand, T2DM causes damage to the BBB, which increases permeability to a variety of substances, and increased leukocyte extravasation, pro-inflammatory cytokine release, and microglia activation occur, resulting in central neuroinflammation and neuronal damage, further disrupting hippocampal function leading to cognitive impairment (Stranahan et al., 2016; Varatharaj and Galea, 2017). In the neuroinflammation process, increased glucose concentration in cells leads to increased oxidative respiration and reactive oxygen species (ROS) production (Wang et al., 2012). Then, ROS reacts with NO to form peroxynitrite. Increased glucose also leads to the formation of advanced glycosylation end products (AGEs), which act on the receptor for advanced glycosylation end products (RAGE) and increase the activation of NF-κB (Ding et al., 2020). Activated NF-κB in turn increases the expression of pro-inflammatory genes, including RAGE and cytokines. VEGF released from astrocytes activates protein kinase C (PKC) and Rho-associated kinase (ROCK), which further promotes inflammation (Van Dyken and Lacoste, 2018).

**FIGURE 13:**
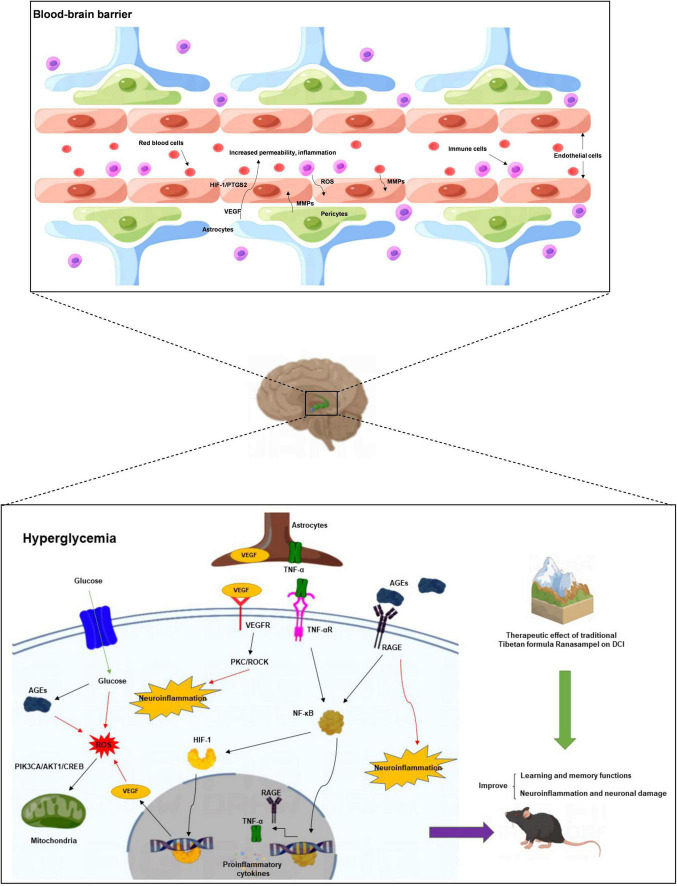
The mechanism of Ranasampel treatment of DCI may be related to the improvement of learning and memory function as well as insulin resistance and hyperglycemia-induced neuronal damage and neuroinflammation.

The Morris water-maze test is primarily used to test the learning and memory abilities of experimental animals (Hernández-Mercado and Zepeda, 2021). In previous studies, it was found that db/db mice had long-term memory impairment (Ye et al., 2018; Ma et al., 2020). In this study, the escape latency of mice in the Ranasampel group was found to be significantly shorter with increasing training days during the 5-day hidden platform trials. In the subsequent probe trial, mice in the Ranasampel group spent significantly more time in the target quadrant than mice in the DCI group. These suggested that Ranasampel improves spatial memory in db/db mice. We also found that Ranasampel attenuated neuronal cell injury and inflammation in the hippocampal CA1, CA3, and DG regions of db/db mice in the histological examination. In the following RNA microarray study, the expressions of 9 hub genes (APP, EP300, FYN, HIF1A, KDR, MMP2, PIK3CA, PPARA, and PTGS2) in the hippocampus were verified. Then, 19 TFs were discovered according to the validated hub genes. It also found that 7 validated TFs (CREB1, CREBBP, ETS2, HIF1A, RELA, SP1, and STAT3) participated in the regulation of APP, EP300, HIF1A, MMP2, PIK3CA, PPARA, and PTGS2. Amyloid precursor protein (APP) is a membrane-intrinsic protein expressed in a variety of tissues and concentrated at the synapses of neurons. The Amyloid beta (Aβ) derived from APP can lead to the formation of Aβ fibers in the brain and neural cell death, which is an important factor in AD (Luu et al., 2021). Impairment of learning and memory abilities is a major manifestation of AD. CREB1, a member of the cAMP response element binding protein family, regulates the transcription of long-term potentiation (LTP)-related genes, which are important for hippocampus-dependent memory (Li Y. et al., 2018). Past work has shown that insulin resistance causes elevated blood glucose and systemic metabolic disturbances, while hyperglycemia stimulates VEGF angiogenesis, leading to immature and unstable blood vessels (Kida et al., 2021). Moreover, VEGF also regulates angiogenesis in response to hypoxia through hypoxia-inducible factor 1 (HIF1) and PTGS2 (Wu et al., 2006; Masoud and Li, 2015). These lead to increased BBB permeability, neuroinflammation, and neuronal damage. HIF1, one of the upstream regulators of VEGF, is also upregulated in response to hyperglycemia and the resulting AGEs and ROS (Catrina, 2014). Downstream, VEGF signaling activates PKC, an enzyme involved in endothelial permeability, then hyperglycemia promotes dysfunction in its pathway, leading to increased NADPH oxidase and MMP2, further promoting cytokine expression and oxidative stress (Shao and Bayraktutan, 2013; Dorfman and Thaler, 2015). Some AGEs can cross-link proteins and produce substrates that inhibit normal protein function. Their production leads to the formation of ROS that causes damage to cells through oxidative stress (Ceriello, 2012; Perrone et al., 2020). ROS can also inhibit the downstream pathway of PIK3CA/AKT1/CREB, leading to mitochondrial function, which in turn weakens the intra-central ROS clearance and impairs neuronal development and nutrition (Ji et al., 2021). In addition, RAGE activates immune response by regulating many cytokines, while many RAGE effects are mediated through NF-κB (Tóbon-Velasco et al., 2014). Activation of NF-κB leads to upregulation of pro-inflammatory cytokines TNF-α, TGF-β1, and IL-1α expression, which enhances leukocyte infiltration and promoted further expression of RAGE (Van Dyken and Lacoste, 2018). Therefore, the inflammatory state induced by hyperglycemia will persist for a long time after the blood glucose level is controlled, and the alleviation of DCI requires effective control of neuroinflammation in addition to hypoglycemia. As shown in our results, the therapeutic effect of Ranasampel on DCI is not only related to its efficacy in improving learning and memory functions in the hippocampus but also associated with the modulation of HIF1A, EP300, MMP2, PIK3CA, PPARA, PTGS2, RELA, SP1, and STAT3 related neuroinflammation and neuronal damage in DCI (Figure 13). Although further studies are needed to determine the underlying mechanisms, our study highlights the potential of the traditional Tibetan formula Ranasampel as an effective treatment for DCI.

## 5 Conclusion

Generally, the current research combined serum pharmacochemistry and bioinformatics approaches to provide numerous testable hypotheses about the molecular mechanisms of the Ranasampel formula for the treatment of DCI. As predicted and verified, Ranasampel treatment improved diabetes mellitus-associated cognitive impairment behaviors and pathological changes in db/db mice and regulated many biological processes (response to oxidative stress, autophagy, learning or memory, cognition, neuron death, etc.) and pathways (VEGF signaling pathway, AGE-RAGE signaling pathway in diabetic complications, HIF-1 signaling pathway, Insulin resistance, TNF signaling pathway, etc.). These results provided a preliminary explanation of the ameliorative effect of Ranasampel against DCI and might precipitate the development of Ranasampel or its active compounds as an alternative therapy for DCI. However, the efficacy of Ranasampel on changes in metabolites and genes requires further evaluation in clinical and basic research, which will be the focus of our future research.

## Data availability statement

The datasets presented in this study can be found in online repositories. The names of the repository/repositories and accession number(s) can be found in the article/Supplementary material.

## Ethics statement

The animal study was reviewed and approved by this study was performed in agreement with the recommendations by the existing current animal welfare guidelines. The animal experimental protocols were approved by the Institutional Animal Care and Use Committee of Beijing University of Chinese Medicine (No. BUCM-4-2022012105-1060).

## Author contributions

ZY, YZ, and TL conceived and designed the study. ZY drafted and wrote the manuscript. YZ, CZ, and ZH carried out the animal experiment. LW and LQ analyzed the data. All authors read and approved the final manuscript.

## Footnotes

1
http://pubchem.ncbi.nlm.nih.gov/

2
http://www.chemspider.com/

3
http://prediction.charite.de/

4
http://www.cytoscape.org/

5
http://www.ncbi.nlm.nih.gov/geo/

6
https://tau.cmmt.ubc.ca/eVITTA/

7
https://tau.cmmt.ubc.ca/eVITTA/easyGEO/

8
https://string-db.org/

9
http://genemania.org/

10
http://en.biocloud.net/

11
http://www.grnpedia.org/trrust/
