# Supplementary material for: Exploring the role of Tibetan medicinal formula Qishiwei Zhenzhu Pills (Ranasampel) against diabetes mellitus-linked cognitive impairment of db/db mice through serum pharmacochemistry and microarray data analysis
Source: Front Aging Neurosci. 2022 Dec 22;14:1033128. doi: 10.3389/fnagi.2022.1033128 (PMC9814129; doi:10.3389/fnagi.2022.1033128)
Supplement: Supplementary file 1 [file Data_Sheet_1.ZIP › Supplementary Table S3.docx]

**Supplementary Table S3.** The top 20 hub genes of 7 algorithms rank in cytoHubba.

| MCC | MNC | EPC | Degree | Closeness | Stress | Radiality |
| --- | --- | --- | --- | --- | --- | --- |
| PPARA | PPARA | PPARA | PRKACA | PRKACA | PRKACA | PRKACA |
| EP300 | EP300 | EP300 | PPARA | PPARA | PPARA | PPARA |
| KDR | KDR | KDR | EP300 | EP300 | EP300 | EP300 |
| AKT2 | AKT2 | AKT2 | KDR | KDR | KDR | KDR |
| MCL1 | APP | MCL1 | AKT2 | AKT2 | ACHE | AKT2 |
| APP | PIK3CA | APP | APP | MCL1 | SLC1A2 | MCL1 |
| PIK3CA | GSK3B | PIK3CA | PIK3CA | APP | APP | APP |
| GSK3B | HSP90AA1 | GSK3B | GSK3B | PIK3CA | PIK3CA | PIK3CA |
| HSP90AA1 | FYN | HSP90AA1 | HSP90AA1 | GSK3B | GSK3B | GSK3B |
| FYN | PTGS2 | FYN | FYN | HSP90AA1 | HSP90AA1 | HSP90AA1 |
| PTGS2 | CHUK | PTGS2 | PTGS2 | FYN | FYN | FYN |
| CHUK | PTPN1 | PTPN1 | PTPN1 | PTGS2 | GRIA2 | PTGS2 |
| PTPN1 | PDGFRB | PDGFRB | PDGFRB | PTPN1 | PTGS2 | PTPN1 |
| PDGFRB | SIRT3 | SIRT3 | SIRT3 | SIRT3 | SIRT3 | SIRT3 |
| MMP2 | MMP2 | MMP2 | MMP2 | MMP2 | MAPK8 | MMP2 |
| MAPK8 | MAPK8 | MAPK8 | MAPK8 | MAPK8 | MMP2 | MAPK8 |
| AR | AR | AR | AR | AR | HIF1A | AR |
| HIF1A | HIF1A | HIF1A | HIF1A | HIF1A | ESR1 | HIF1A |
| ESR1 | ESR1 | ESR1 | ESR1 | ESR1 | PRKCA | ESR1 |
| PRKCA | PRKCA | PRKCA | PRKCA | PRKCA | FASN | PRKCA |
